# 

**Published:** 1875-08

**Authors:** 


THE INFLUENCE OF THE CLIMATE OF COLORADO on the Nervous System. By Charles Dennison, M.D., Denver, Colorado. Reprinted from the Archives of Electrology and Neurology. 1875. Pamphlet; pp. 16.

---

THE EXTENSIOR WINDLASS, Presented to the American Medical Association, May, 1875. By Charles Dennison, M.D., Denver, Colorado. Reprinted from the *New York Medical Journal.* 1875. Pamphlet; pp. 11.

---

SAVANNAH MEDICAL COLLEGE. Circular and Catalogue of the Trustees, Faculty and Alumni. Announcement of Lectures, Session 1875–76. With an Address by A. P. Adams, Esq., delivered at the Commencement, March, 1875. Pamphlet; pp. 21.

